# An Assessment of the Antifungal Efficacy of a Novel Topical Onychomycosis Treatment Using Human Nail and Skin Infection Models

**DOI:** 10.3390/jof11050345

**Published:** 2025-04-29

**Authors:** Anthony Brown, Felipe Goñi-de-Cerio, Ainhoa Bilbao, Adrià Ribes, Antonio R. Fernández de Henestrosa, Ludmila Prudkin, Paola Perugini, Mónica Foyaca

**Affiliations:** 1 Innovation and Development, ISDIN, 08019 Barcelona, Spain; 2 GAIKER Technology Centre, Basque Research and Technology Alliance (BRTA), 48170 Zamudio, Spain; 3 Department of Pharmaceutical Chemistry, University of Pavia, 27100 Pavia, Italy

**Keywords:** onychomycosis, *Trichophyton rubrum*, *Epidermophyton floccosum*, *Aspergillus versicolor*, *Fusarium solani*, *Candida albicans*, ex vivo model, topical, antifungal, nail

## Abstract

Onychomycosis, a fungal nail infection, affects about 4% of the global population. Current topical antifungals like ciclopirox and amorolfine have limited effectiveness, highlighting the need for better treatments. WSNS-PO is a novel water-soluble therapy designed to treat and prevent onychomycosis by enhancing nail health. This study evaluated WSNS-PO’s ability to penetrate the nail plate and to treat and prevent infection by *Trichophyton rubrum* using bovine hoof membranes and human nail clippings. The anti-fungal efficacy of WSNS-PO was additionally evaluated against other dermatophytes, non-dermatophyte fungi, and yeast. The results showed that WSNS-PO effectively permeated nails and reduced and prevented the colonization of human nail fragments by *T. rubrum* ex vivo, demonstrating an efficacy comparable to ciclopirox and amorolfine. WSNS-PO also prevented the transfer of *T. rubrum* infection between nails and inhibited the fungal colonization of human skin by dermatophyte and non-dermatophyte fungi and yeast. Together, these results indicate that WSNS-PO possesses fungistatic, barrier-forming, and anti-adhesive properties, suggesting that it holds promise as an onychomycosis treatment against dermatophytes, yeast, and molds.

## 1. Introduction

Onychomycosis is a chronic fungal infection that affects the toenails or fingernails, leading to discoloration, thickening of the nail plate, and separation of the nail from the nail bed. The infection is caused by dermatophytes, non-dermatophyte molds (NDMs), and yeasts. Most infections are caused by dermatophyte fungi, such as *Trichophyton rubrum*, *Trichophyton mentagrophytes*, and *Epidermophyton floccosum*. However, infections caused by yeasts, such as *Candida* species, and NDMs, such as *Aspergillus* species, are becoming more common [1,2]. Mixed infections of dermatophytes and NDMs are estimated to account for up to 40% of onychomycosis cases [3].

Onychomycosis is difficult to treat and often results in high rates of recurrence and treatment failure. Many infections are self-diagnosed, with individuals initially self-treating using over the counter medications [4]. Topical treatments, such as nail lacquers containing 5% amorolfine and 8% ciclopirox, are commonly used for mild to moderate onychomycosis [5]. However, these treatments require frequent application over extended periods to achieve a complete cure (i.e., a fully clear nail with a negative culture and microscopy). Additionally, the cosmetically unappealing nature of nail lacquers, combined with the need to frequently remove previous applications for better penetration of subsequent doses [6,7], negatively impacts treatment adherence. As a result, mycological and complete cure rates with these agents are often low, highlighting the urgent need for alternative treatments. Currently, however, most research is focused on discovering new antifungal agents or enhancing the effectiveness of existing ones using penetration enhancers [7]. Few studies explore alternative methods for treating or preventing fungal infections.

A water-soluble cosmetic nail strengthener (WSNS) has recently been shown to improve the physical properties and cosmetic appearance of nails [8,9]. This WSNS contains a blend of ingredients designed to support the natural process of nail growth [8,9]. Hyaluronic acid helps boost the hydration of the nail plate, cuticle, and surrounding periungual skin [9]. Pistacia lentiscus gum (mastic oil), an aromatic resin from the *Pistacia lentiscus* plant, has been shown to stimulate the synthesis of hard keratins (K31, K83, and K85) in vitro [9]. Additionally, silanediol salicylate, a source of biological silicon, supports the growth of the nail and surrounding periungual tissues [9]. WSNS was specifically developed to be effective, cosmetically appealing, and easy to use [8]. During clinical evaluations, it was observed that WSNS could also improve the appearance of nails in individuals suffering from onychomycosis. Based on these findings, we hypothesized that WSNS might also be an effective treatment for onychomycosis. As a result, we developed a new medical device based on WSNS but supplemented with piroctone olamine (WSNS-PO) to ensure the sterility of WSNS during its repeated use on infected nails.

The aim of this study was to assess whether WSNS-PO could play a role in the prevention and management of onychomycosis by evaluating its efficacy in experimental models of onychomycosis.

## 2. Materials and Methods

### 2.1. Test Products

The investigative product (WSNS-PO) is a hydrosoluble nail lacquer containing piroctone olamine (PO) in combination with active ingredients designed to support nail growth (i.e., silanediol salicylate, *Pistacia lentiscus* gum, and cationic hyaluronic acid). It is based on a formulation (WSNS) that has been described previously [10,11]. Ciclopoli^®^ (CIC), a marketed formulation containing 8% ciclopirox, was obtained from a German pharmacy. Amorolfina Isdin 50 mg/mL (AMOR) was donated by the study sponsor.

### 2.2. Minimal Inhibitory Percentage (MIP)

The Minimal Inhibitory Percentage (MIP), an adaptation of the Minimum Inhibitory Concentration at 100% inhibition (MIC_100_), was used to evaluate the antifungal activity of multi-component topical formulations, such as WSNS-PO and WSNS. MIP, defined as the lowest concentration (expressed as a percentage) of a product that visibly inhibits microbial growth under testing conditions simulating topical product use, was determined based on the CLSI M38-A2 protocol with modifications. Briefly, 100 μL of WSNS-PO, WSNS, CIC, and AMOR were added to each well of a 96-well plate, and serial dilutions were performed to obtain a final product concentration of 0.001%. Then, 100 μL of a suspension of *T. rubrum* ATCC 28188 (1 × 10^6^ conidia/mL in Sabouraud (SAB) medium, prepared using a hemocytometer) was added to each well, resulting in a final inoculum of 1 × 10^5^ conidia per well. Plates were incubated at 25 °C for 7 days to monitor fungal growth. A total of 100 μL of media from the wells where fungal growth was not observed was subsequently inoculated in Petri dishes with SAB agar. Petri dishes were incubated at 25 °C for 7 day, and fungal growth was monitored. The MIP was the lowest concentration of product that inhibited the growth of *Trichophyton*. Three replicates at each concentration were performed.

### 2.3. Agar Disc Diffusion Assay

SAB agar plates were seeded with 5 × 10^7^ CFU/mL *T. rubrum* ATCC 28188. WSNS-PO, WSNS, CIC, and AMOR were then infused into small filter paper discs and subsequently placed on the surface of the agar plate. Plates were incubated at 25 °C and the zone of inhibition relative to the total growth area of the fungus was measured after 7 days. Results were expressed as a mean ± Standard Deviation (SD) of 3 experiments.

### 2.4. Permeation Through Bovine Hoof Membranes

Membranes from bovine hooves of about 400 μm thickness were harvested from freshly slaughtered cows obtained from an abattoir. Following characterization for morphology and thickness, membranes were sterilized by washing in ethanol 70% *v*/*v* and a benzalkonium chloride mixture (0.4 g Benzalkonium Chloride, 70 g isopropyl alcohol, distilled water to 100 g). Sterilized membranes were then treated with WSNS-PO, AMOR, and CIC (60 mg per application) for 5 days. During this preloading phase, all membranes were maintained in a climatic chamber at 20 °C and 40% relative humidity (RH).

Treated membranes were then placed on SAB agar plates seeded with *T. rubrum* ATCC 28188 and incubated in a climatic chamber at 24 °C and 40% RH. Membranes were treated with products for an additional five days and the zone of inhibition relative to the total growth area of the fungus measured after 7 days. Results were expressed as a mean ± SD of 3 experiments.

### 2.5. Ex Vivo Experimental Infection by T. rubrum

A culture of *T. rubrum* CECT 2794 (provided by CECT—Colección Española de Cultivos Tipo) was used for all ex vivo infection studies. Briefly, distal nail fragments were obtained from healthy, 40- to 65-year-old women undergoing aesthetic manicures and pedicures. To prepare nails for use, nails were first autoclaved at 121 °C for 20 min and thereafter manually cut into fragments of equal size (0.2 cm^2^). To induce infection, 500 μL of a *T. rubrum* suspension (1–5 × 10^7^ CFU/mL) was incorporated into nutrient-free Yeast Nitrogen Base agar (YNB). Sterilized healthy nail fragments were then placed onto the inoculated medium and incubated at 25 °C for 1 week with daily inspection.

#### 2.5.1. Evaluation of Curative Activity

**Figure 1:**
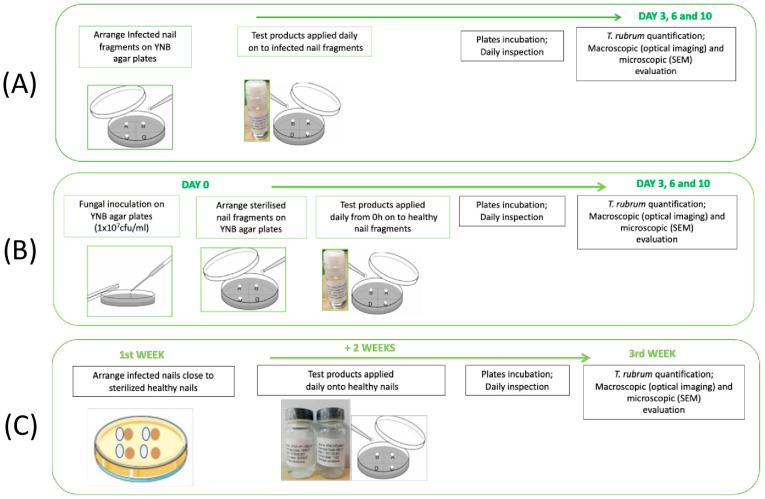
Schematic representation of protocol employed for (**A**) evaluation of curative activity, (**B**) evaluation of preventive activity, and (**C**) evaluation of capacity to prevent cross-infection.

Infected nail fragments were transferred to new YNB agar plates and 2 μL of WSNS-PO, AMOR, and CIC was applied to the surface of infected nails once per day for 10 days. Treated nails were then incubated at 25 °C. Viable fungal cell recovery from nails was performed on days 3, 7, and 10 by tape-stripping. Following recovery, tapes were placed in Phosphate Buffer Solution (PBS) + 0.1% Triton X-100 and the resulting suspension was applied to glucose chloramphenicol agar plates following serial dilutions. Plates were incubated at 25 °C for 48–72 h and colony forming units (CFUs) were subsequently counted. Six replicates per experimental group were performed (five replicates for fungal quantification, and one replicate for determination of the presence of fungus by macroscopic and microscopic evaluation). A non-treated nail was used as a control. A schematic representation of the protocol is given in Figure 1A.

#### 2.5.2. Evaluation of Preventive Activity

WSNS-PO, AMOR, and CIC (2 μL per application) were first applied to the surface of uninfected healthy nails. Treated nails were then placed on YNB agar plates that had previously been inoculated with *T. rubrum*. Treatment was repeated once per day for 10 days. Viable fungal cells recovery and CFU counts were performed on days 3, 7, and 10 as described above. Six replicates per experimental group were performed (five replicates for fungal quantification, and one replicate for determination of the presence of fungus by macroscopic and microscopic evaluation). A non-treated nail was used as a control. A schematic representation of the protocol is given in Figure 1B.

#### 2.5.3. Evaluation of Capacity to Prevent Cross-Infection

Briefly, infected nail fragments were transferred to YNB agar plates, where they were placed adjacent to healthy (uninfected) ones. Treatment was then commenced by daily application of WSNS-PO (2 μL per application) to the uninfected nails for an additional 2 weeks. Following treatment, viable fungal cell recovery and CFU counts were performed as described above. Six replicates per experimental group were performed (five replicates for fungal quantification, and one replicate for determination of the presence of fungus by macroscopic and microscopic evaluation). A non-treated nail was used as a control. A schematic representation of the protocol is given in Figure 1C.

#### 2.5.4. Scanning Electron Microscopy

Briefly, ex vivo nails were fixed in a 2% glutaraldehyde solution. Subsequently, nail samples were incubated in cacodylate + isomolar sucrose and finally dehydrated with serial ethanol solutions. Nail samples were then coated with gold under an argon atmosphere. Scanning electron microscopy was performed using a Hitachi S-4800 FEG Scanning Electron Microscope (Hitachi Ltd., Tokyo, Japan).

### 2.6. Antifungal Against Dermatophyte and Non-Dermatophyte Fungi and Yeasts

Normal human organotypic skin explant cultures were used in all studies. Residual skin following abdominoplasty was obtained with informed consent, under authorization granted by the French government ethical committee according to French law L.1245 CSP, from healthy, 40- to 55-year-old women undergoing plastic surgery. Within 2 h of surgery, skin was cut into 0.8 cm^2^ pieces and placed dermis side down in culture plates containing Dulbecco’s Modified Eagle’s Medium (DMEM) containing antibiotics (1% penicillin/streptomycin). Cultures were incubated for 48 h at 37 °C under 5% CO_2_ for recovery prior to study initiation.

All fungi strains used in this study were obtained from publicly available collections: The Leibniz Institute DSMZ—German Collection of Microorganisms and Cell Cultures GmbH, CCUG—Culture Collection University of Gothenburg, and CECT—Colección Española de Cultivos Tipo. The strains included *Candida albicans* DSMZ 11948, *Trichophyton rubrum* CECT 2794, *Trichophyton interdigitale* CECT 2921, *Epidermophyton floccosum*, *Aspergillus versicolor*, and *Fusarium solani*.

Briefly, WSNS-PO was topically applied to the surface of skin explants using a micropipette and subsequently spread using a microspatula to ensure homogenous application of 10 µL of WSNS-PO per cm^2^. Following conditioning for 24 h, 10 µL of each fungal suspension (5 × 10^5^–5 ×10^6^ CFU) was applied to the surface of the skin and incubated for an additional 24 h at 37 °C and 5% CO_2_. Five replicates per experimental group (four replicates for antifungal functional barrier activity assay and one replicate for Gram staining) were performed. Negative (explant without WSNS-PO or fungus) and positive (fungus without WSNS-PO) controls were also included.

Viable fungal cell recovery from skin explants was conducted by tape-stripping followed by dilution in PBS + 0.1% Triton X-100. The resulting suspension was then applied to glucose chloramphenicol agar plates. Plates were then incubated at 30 °C for 48–72 h and CFU counts were determined.

### 2.7. Statistical Analyses

Values are given as mean ± SD. The homogeneity of variance was confirmed by the Levene test, and the normality was confirmed by the Anderson–Darling test. Unpaired *t*-tests and one factor analysis of variance (ANOVA) with Bonferroni–Dunn’s correction were conducted to assess differences in fungal CFU with respect to an untreated infected nail control group. A *p* < 0.05 was considered significant. Log differences between treated and untreated nails were additionally calculated. Where no colonies were detected (ND), a value of 20 was used for all calculations.

## 3. Results

### 3.1. Minimal Iinhibitory Percentage (MIP)

The results obtained from MIP analysis (Table 1) demonstrated that WSNS-PO and an equivalent formulation without PO (WSNS) show an inhibitory activity against *T. rubrum* ATCC 28188 at a concentration equal to 50%, the maximum concentration assessed. CIC shows an inhibitory action at a concentration equal to 25%, while AMOR is significantly more active, showing an inhibitory action at a concentration equal to 1.56%.

### 3.2. Agar Disc Diffusion Assay

Both CIC and AMOR exerted maximum activity, with 100% inhibition of *Trichophyton* spp. growth. WSNS-PO and WSNS demonstrated comparable activity, inhibiting 52.23 ± 6.34% and 49.72 ± 9.90% of fungal growth, respectively (Table 1).

### 3.3. Permeation Study Through Bovine Hoof Membranes

The permeation results (Table 1) show that the control membrane without any treatment only caused a minimum halo (3.34 ± 4.72% inhibition), while a solution of 50% ethanol demonstrated limited effectiveness by inhibiting 12.13% of the plate area. AMOR was most effective and able to completely inhibit the growth of *T. rubrum* ATCC 28188 on the entire surface (100% inhibition). CIC reduced the growth area of *T. rubrum* ATCC 28188 by 84.24 ± 13.65%, while WSNS-PO reduced it by 63.85 ± 32.17%.

### 3.4. Curative Activity Against Experimental T. rubrum Infection

All tested products demonstrated very good efficacy in terms of reduction in viable fungal cells in nails previously infected with *T. rubrum*, with a significant (*p* < 0.05) reduction of more than 3.47 log of *T. rubrum* CFU compared to the untreated group after 3 days of treatment. Efficacy was even greater after 6 and 10 days of treatment, with all test products reducing *T. rubrum* CFUs by more than 4.81 and 5.43 log, respectively (*p* < 0.01), compared to the untreated group (Table 2). Conversely, in untreated control nails, *T. rubrum* infection continued to develop throughout the 10-day duration of this study.

**Figure 2:**
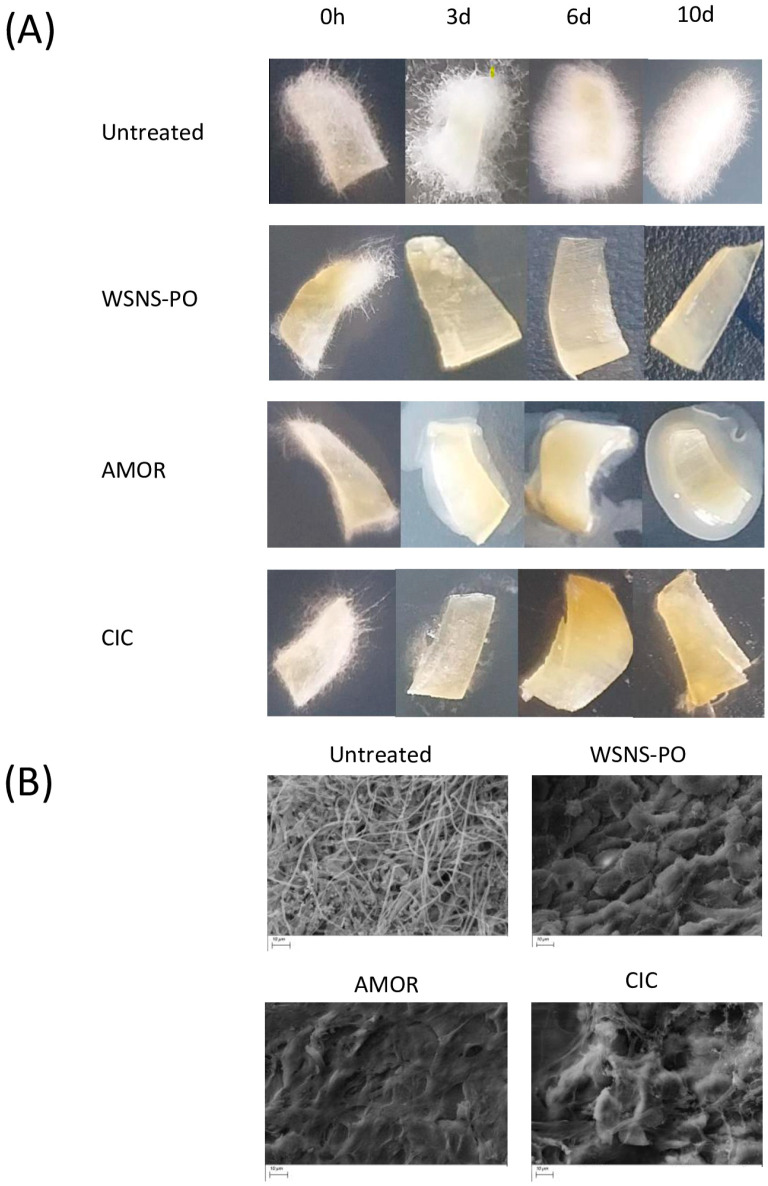
Macro- and microscopic aspect of nail fragments from infection elimination (curative activity) study. (**A**) Macroscopic images of infected nail fragments following incubation for (from left to right) 0 h, 3 days, 6 days, and 10 days: untreated control nail (top), WSNS-PO-treated nail (second row), AMOR-treated nail (third row), and CIC-treated nail (bottom). (**B**) Magnified views (×2.00 K) obtained with SEM following 10 days of treatment: untreated control nail (top left), WSNS-PO-treated nail (top right), AMOR-treated nail (bottom left), and CIC-treated nail (bottom right). Scale bars represent 10 µm.

The SEM analysis demonstrated that *T. rubrum* efficiently penetrated the nail fragments in untreated nails. Fungal hyphae can be seen covering the entire surface of the nail and penetrating it. It also confirmed that *T. rubrum* infection persisted after 10 days on the surface of untreated control nails (Figure 2B) in accordance with the macroscopic evaluation (Figure 2A). Conversely, daily application of the products for 10 days resulted in a reduction in fungal hyphae as evidenced microscopically (Figure 2B). Although traces of fungus are observed on the nail surfaces, CFU quantification (Table 2) confirmed that *T. rubrum* was not detected after 10 days of treatment with the tested products.

### 3.5. Preventative Activity Against Experimental T. rubrum Infection

**Figure 3:**
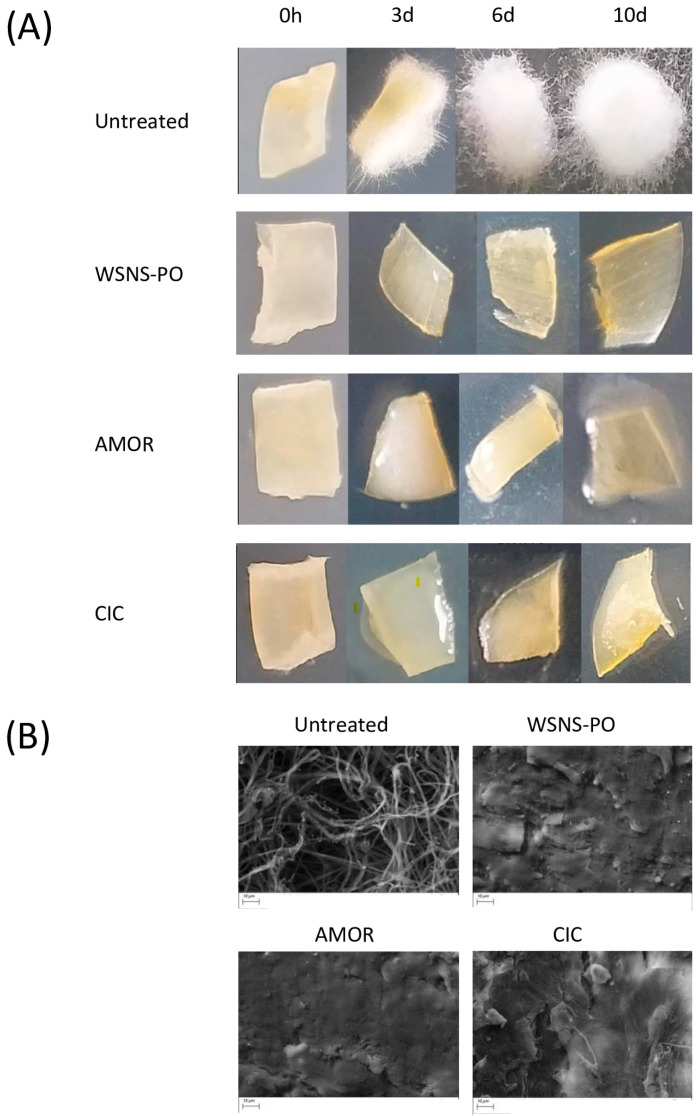
Macro- and microscopic aspect of nail fragments from infection prevention study. (**A**) Macroscopic images of nail fragments following incubation for (from left to right) 0 h, 3 days, 6 days, and 10 days: untreated control nail (top), WSNS-PO-treated nail (second row), AMOR-treated nail (third row), and CIC-treated nail (bottom). (**B**) Magnified views (×2.00 K) obtained with SEM following 10 days of treatment: untreated control nail (top left), WSNS-PO-treated nail (top right), AMOR-treated nail (bottom left), and CIC-treated nail (bottom right). Scale bar represents 10 µm.

Uninfected nails placed on agar inoculated with *T. rubrum* and treated with the tested products showed a very good efficacy in terms of prevention of onychomycosis infection from day 3 onwards. A significant difference (*p* < 0.05) in *T. rubrum* infection was observed between the untreated and treated groups, with a log difference of more than 3.76 and 5.39 observed on days 6 and 10, respectively (Table 2). These quantitative results were confirmed by a macroscopic and microscopic (SEM) evaluation of the ex vivo nails (Figure 3). Conversely, *T. rubrum* infection continued to develop on the surface of untreated control nails during the 10 days of the study, with visible fungal hyphae covering the surface of the nail and penetrating it (Figure 3).

### 3.6. Prevention of Transfer of Nail Infection

**Figure 4:**
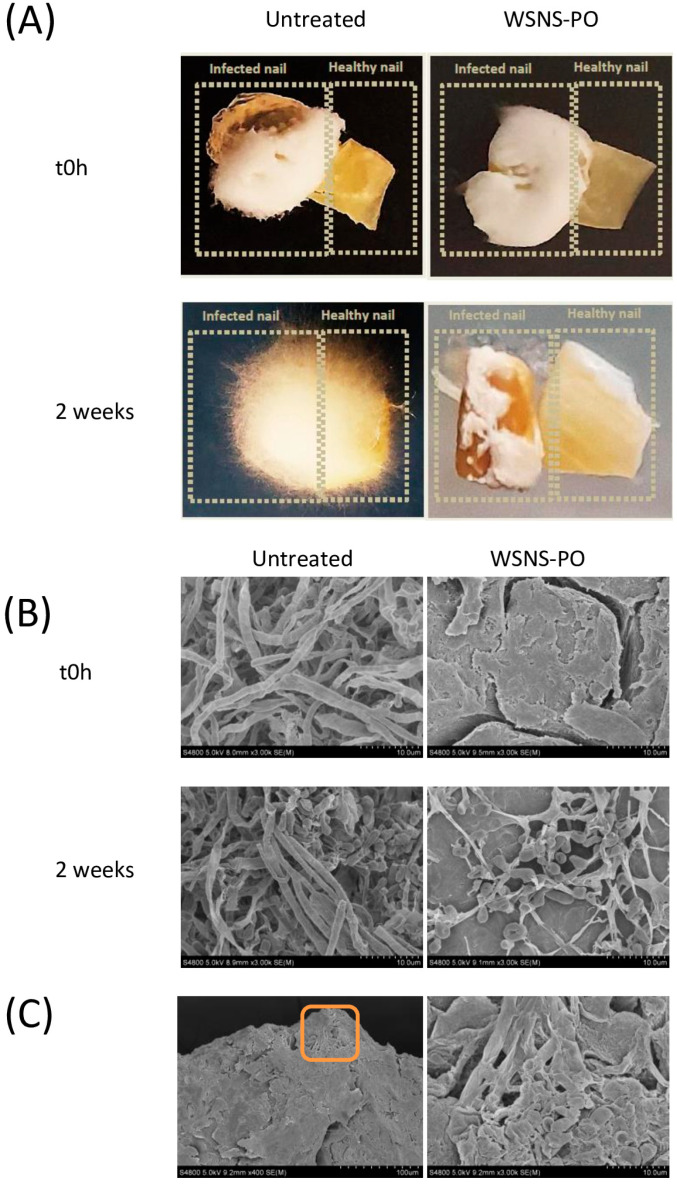
Macro- and microscopic aspect of nail fragments from transfection prevention study. (**A**) Macroscopic images of control (left) and WSNS-PO-treated nail fragments (right) at time 0 h (top) and after 2 weeks of treatment (bottom). (**B**) Magnified views (×3.00 K) obtained with SEM of control (left) and WSNS-PO-treated nail fragments (right) at time 0 h (top) and after 2 weeks of treatment (bottom). Scale bar represents 10 µm. (**C**) Microscopic aspect of fungal hyphae trying to invade an ex vivo nail fragments treated with WSNS-PO. Magnified view (×400) obtained with SEM (left). The right pane shows a magnified view (×3.00 K) of the orange box.

As shown in Figure 4, ex vivo transfection from an infected nail to a healthy untreated nail successfully occurs during the 2-week duration of this study. WSNS-PO was highly effective at preventing transfection between nails, with 4.98 log fewer *T. rubrum* detected in treated healthy nails compared to the non-treated group (*p* < 0.001; Table 3). These quantitative results were confirmed by macroscopic and microscopic (SEM) evaluation of ex vivo nails (Figure 4). Macroscopically, no transfection was observed on the treated healthy nail (Figure 4A). Interestingly, untreated infected nail fragments also exhibited a 1.5 log reduction in *T. rubrum* (Table 3), with nails exhibiting areas devoid of fungus on their surface (Figure 4A), suggesting that the product diffused from the treated nail to the untreated nail during the course of this study. SEM analysis also confirmed this.

Figure 4C shows the contact point (at different magnifications) of both nails (infected control and uninfected treated nails), revealing how *T. rubrum* unsuccessfully attempted to pass from the infected nail to the non-infected treated nail at their points of contact.

### 3.7. Antifungal Against Dermatophyte and Non-Dermatophyte Fungi and Yeasts

WSNS-PO prevented the colonization of skin ex vivo by dermatophyte *T. rubrum*, *T. interdigitale*, *E. floccosum*, and *C. albicans* with a log difference greater than 2.5 observed between treated and untreated groups (Table 4). WSNS-PO was less effective against the NDM *A. versicolor* (1.73 log reduction) and *F. solani* (0.58 log reduction) (Table 4).

## 4. Discussion

These studies demonstrate that WSNS-PO may be an effective treatment for onychomycosis. In an ex vivo onychomycosis model, WSNS-PO exhibited strong activity against *T. rubrum*, the dermatophyte responsible for over 80% of infections. It nearly eradicated fungal infection from infected nail clippings and was equally effective in preventing colonization in uninfected nails. Additionally, using a novel transfection model, we show that WSNS-PO can prevent cross-infection between nails. Given the high transmissibility of onychomycosis, this finding is particularly significant. To our knowledge, this is the first study to evaluate this aspect of treatment efficacy.

In the bovine hoof membrane model, which assesses a product’s ability to penetrate the nail and inhibit fungal growth in an underlying layer, WSNS-PO exhibited a lower efficacy than AMOR and CIC, suggesting that WSNS-PO may have lower nail penetration compared to these antifungals. However, given that WSNS-PO’s MIP is twice that of CIC and over 32 times higher than AMOR, this difference likely reflects its inherently lower fungistatic potential. This is unsurprising, as PO was included to maintain formulation sterility, and its antifungal effects are clearly secondary to the formulation’s overall efficacy. Notably, in the ex vivo nail infection model, WSNS-PO demonstrated an equivalent efficacy to AMOR and CIC, suggesting it exerts its antimycotic effects through an alternative mechanism. These findings underscore the importance of evaluating treatments across multiple experimental models, particularly for products like WSNS-PO that do not rely solely on direct fungicidal activity.

The MIP and zone of inhibition testing revealed that WSNS-PO and its equivalent formulation without PO (WSNS) exhibit comparable antifungal activity. MIP, an approach that is conceptually similar to the Minimal Inhibitory Concentration (MIC) conventionally used for the microbial susceptibility testing of pure compounds, was adapted here to assess the antimycotic efficacy of the complex WSNS-PO formulation under conditions that more closely reflect real-world topical product use (i.e., lower temperatures and a nutrient-rich medium). Furthermore, although agar disc diffusion is not typically employed for dermatophytes due to their slow growth and inconsistent diffusion patterns, its use in this study effectively highlights the limited antifungal activity of WSNS-PO.

Since WSNS lacks any conventional antifungal drug, the comparable efficacy of WSNS and WSNS-PO suggests that at least one of its components possesses inherent fungicidal/fungistatic properties. Although we have not individually assessed each ingredient for antifungal activity, previous studies have reported that gum and leaf extracts of *Pistacia lentiscus* exhibit fungicidal effects against *T. mentagrophyte*, *T. rubrum*, and *C. albicans* [12,13]. The primary constituent of Pistacia lentiscus gum is resin, which contains approximately 2% volatile oil, predominantly α-D-Pinene [14], a compound shown to exert antimycotic activity against *C. albicans* at millimolar concentrations [15].

WSNS-PO also effectively prevented the colonization of human skin ex vivo by dermatophytes (*T. rubrum*, *T. interdigitale*, and *E. floccosum*), yeast (*C. albicans*), and non-dermatophyte molds (*F. solani* and *A. versicolor*), suggesting broad-spectrum activity against the major fungal pathogens responsible for onychomycosis. The findings from this study, along with ex vivo prevention and transfection experiments, suggest that WSNS-PO forms a stable film that acts as a protective barrier against infection by inhibiting fungal adhesion and preventing the formation of incipient hyphae. In addition to Pistacia lentiscus gum, which is known for its excellent film-forming properties [14], WSNS-PO contains a rhamnose-rich polysaccharide (Biosaccharide-gum2) that binds strongly to keratinocyte receptors, reducing microbial adhesion to the skin surface [16]. In human skin explants, WSNS-PO significantly inhibited the adhesion of dermatophyte fungi (*T. rubrum*, *T. interdigitale*, and *E. floccosum*) and, to a lesser extent, non-dermatophyte molds (*F. solani* and *A. versicolor*), suggesting that this polysaccharide may interact with epitopes on keratin chains. Targeting the early stages of infection by inhibiting microbial adhesion is an emerging strategy for the prevention and treatment of bacterial and fungal infections [17,18].

Onychomycosis is a condition that not only affects the nail plate but also frequently extends to the surrounding nail bed and periungual skin. The nail bed and surrounding skin are critical sites for fungal colonization and infection recurrence. Nail fragments, being composed primarily of dead, keratinized tissue, cannot replicate the living tissue environment of the nail bed, where significant interactions between the fungus, host immune system, and therapeutic agents occurs. In contrast, skin explants maintain viable tissue, providing an ex vivo model that closely mimics the infection dynamics in vivo. The inclusion of skin explants in this study allows for a more accurate representation of fungal invasion from the nail into deeper tissues, such as the nail bed, where pathogens can evade treatment or cause a relapse. This is especially important because effective treatment requires not only antifungal action at the nail surface but also the ability to penetrate the underlying tissues. This combined approach ensures that WSNS-PO’s performance is thoroughly assessed not only on the nail itself but also in the surrounding living tissues, providing a more accurate and pre-clinically relevant model for onychomycosis treatment.

Another potential mechanism through which WSNS-PO acts is by hydrating the nail plate. It achieves this in two main ways. First, its film-forming properties help reduce transonychial water loss (TOWL). Second, WSNS-PO contains hyaluronic acid (HA), which further boosts hydration of the nail plate [9]. Water is a key plasticizer for nails, and when the nail becomes hydrated, it gains elasticity and becomes more permeable to topically applied substances [19]. The positive charge of HA also gives WSNS-PO a strong adhesive ability, helping it stay on the skin and nails longer [8]. Additionally, increased hydration of the nail plate may reduce the reservoir of dormant conidia within, potentially limiting the formation and persistence of drug-resistant fungal spores [10]. This is particularly important as superficial mycoses are becoming more resistant to current antifungal treatments [11]. Therefore, treatments like WSNS-PO, which act through alternative mechanisms, are valuable as they could help slow the rise of drug-resistant onychomycosis and extend the effectiveness of existing therapies.

Another important benefit of WSNS-PO is its ability to improve both the cosmetic appearance and physical properties of the nail plate. Previous studies have shown that WSNS increases the firmness of bovine hoof membranes and significantly improves nail hardness in individuals with brittle nails [8,9]. These improvements occur quickly: visible changes to the nails can be seen in as little as 2 weeks, and significant increases in nail thickness and density are observed within 1 month [8]. This makes WSNS-PO not only an effective treatment for infection, but also one that rapidly enhances the visual and physical qualities of the nails. Such improvements can help boost clinical cure rates and are likely to improve treatment adherence, which is crucial since poor compliance is a major factor in relapse and reinfection [20,21].

This study has several limitations. First, while in vitro and ex vivo models can be predictive of clinical efficacy, the mycological cure rates observed in these models do not always directly translate to the same results in vivo. Therefore, a clinical confirmation of efficacy is essential before recommending any new treatment for onychomycosis. Clinical trials involving WSNS-PO are currently ongoing.

Another limitation of this study is the sensitivity of the method used to detect *T. rubrum* colonization of the nail plate. In this study, tape-stripping was employed to detect infection. While this method is likely effective at identifying fungi on the surface of the nail, it may not capture fungi that have penetrated the nail plate itself. Although the SEM analysis indicated that no viable fungi were present in nails treated with WSNS-PO, it would be beneficial to supplement this analysis with other techniques, such as measuring ATP production to assess metabolic activity [22,23].

In this study, the antimycotic activity of WSNS-PO in the ex vivo nail infection model was only tested against *T. rubrum*. Future research examining its effectiveness against other species of dermatophytes, NDMs, and molds would be valuable.

In conclusion, the studies conducted in various preclinical models indicate that WSNS-PO effectively prevents and eliminates infections caused by dermatophyte fungi, yeast, and NDMs. This is achieved through a combination of its fungistatic, barrier-forming, and anti-adhesive properties. Given that WSNS-PO demonstrates efficacy that is at least equivalent to that of ciclopirox and amorolfine—while also being more cosmetically acceptable, having a distinct mechanism of action, and improving the visual appearance of infected nails—it may represent a promising treatment for onychomycosis.

## Figures and Tables

**Table 1:** MIP and inhibition area (%) against *Trichophyton* spp. for each product evaluated.

Product	MIP	Paper Disc Zone of Inhibition (% Versus Control ± SD)	Bovine Hoof Membrane Zone of Inhibition (% Versus Control ± SD)
WSNS-PO	50.00%	52.23 ± 6.34	63.85 ± 32.22
WSNS	50.00%	49.72 ± 9.90	-
CIC	25.00%	100.0 ± 0.00	84.24 ± 13.65
AMOR	1.56%	100.0 ± 0.00	100.0 ± 0.00
Control	-	0.00 ± 0.00	3.34 ± 4.72
Ethanol 50%	-	-	12.13 ± 0.00

**Table 2:** CFU quantification for curation and prevention of experimental *T. rubrum* infection. Log difference versus untreated nails is given in parentheses.

Curation	Untreated	WSNS-PO	CIC	AMOR
3 d	5.97 ± 5.14 × 10^4^	ND (>3.47) *	ND (>3.47) *	ND (>3.47) *
6 d	1.38 ± 1.19 × 10^6^	ND (>4.81) **	ND (>4.81) **	ND (>4.81) **
10 d	5.40 ± 0.83 × 10^6^	ND (>5.43) ***	ND (>5.43) ***	ND (>5.43) ***
**Prevention**				
3 d	317 ± 21	ND (>1.2) ***	ND (>1.2) ***	ND (>1.2) ***
6 d	1.16 ± 0.55 × 10^5^	ND (>3.76) *	ND (>3.76) *	ND (>3.76) *
10 d	4.96 ± 1.55 × 10^6^	ND (>5.39) ***	ND (>5.39) ***	ND (>5.39) ***

ND, Not detected; *** *p*-value < 0.001; ** *p*-value < 0.01; * *p*-value < 0.05.

**Table 3:** CFU quantification for prevention of experimental *T. rubrum* transfection. Log difference versus untreated nails is given in parentheses.

	Infected Nail	Healthy Nail
Untreated	2.82 ± 2.77 × 10^6^	2.01 ± 2.71 × 10^6^
Treated	2.00 ± 2.62 × 10^5^ (1.5) ^n.s^.	21 ± 2 (4.98) ***

*** *p*-value < 0.001; n.s., not significant.

**Table 4:** Fungal quantification of skin explants. Log reduction versus untreated skin is given in parentheses.

Species	Untreated ± SD	WSNS-PO ± SD (% Reduction)
*T. rubrum*	2.15 ± 0.27 × 10^5^	88 ± 7807 (3.39) ***
*T. interdigitale*	5.38 ± 1.62 × 10^4^	142 ± 285 (2.58) **
*E. floccosum*	1.54 ± 0.10 × 10^5^	ND (>3.89) ***
*C. albicans*	1.03 ± 0.15 × 10^7^	8515 ± 12,361 (3.08) ***
*F. solani*	9.62 ± 2.04 × 10^4^	2.54 ± 1.31 × 10^4^ (0.58) **
*A. versicolor*	2.87 ± 0.53 × 10^6^	5.34 ± 0.4 × 10^4^ (1.73) ***

ND, Not detected; *** *p*-value < 0.001; ** *p*-value < 0.01.

## Data Availability

The original contributions presented in this study are included in the article. Further inquiries can be directed to the corresponding author.

## Abbreviations

The following abbreviations are used in this manuscript:

WSNS	Water-soluble cosmetic strengthener
PO	Piroctone olamine
NDMs	Non-dermatophyte molds
AMOR	Amorolfine
CIC	Ciclopirox
MIP	Minimum Inhibitory Percentage
RH	Relative humidity
CFM	Colony forming units
SEM	Scanning electron microscopy
SD	Standard deviation
HA	Hyaluronic acid
